# Marine-Derived Polysaccharide Hydrogels as Delivery Platforms for Natural Bioactive Compounds

**DOI:** 10.3390/ijms26020764

**Published:** 2025-01-17

**Authors:** Fabrizia Sepe, Anna Valentino, Loredana Marcolongo, Orsolina Petillo, Raffaele Conte, Sabrina Margarucci, Gianfranco Peluso, Anna Calarco

**Affiliations:** 1 Research Institute on Terrestrial Ecosystems (IRET), CNR, Via Pietro Castellino 111, 80131 Naples, Italy; 2 National Biodiversity Future Center (NBFC), 90133 Palermo, Italy; 3 Faculty of Medicine and Surgery, Saint Camillus International University of Health Sciences, Via di Sant’Alessandro 8, 00131 Rome, Italy

**Keywords:** marine-derived hydrogels, antibacterial properties, bioactive compounds, biocompatibility, sustainable materials, drug delivery systems, biodegradability

## Abstract

Marine polysaccharide hydrogels have emerged as an innovative platform for regulating the in vivo release of natural bioactive compounds for medical purposes. These hydrogels, which have exceptional biocompatibility, biodegradability, and high water absorption capacity, create effective matrices for encapsulating different bioactive molecules. In addition, by modifying the physical and chemical properties of marine hydrogels, including cross-linking density, swelling behavior, and response to external stimuli like pH, temperature, or ionic strength, the release profile of encapsulated bioactive compounds is strictly regulated, thus maximizing therapeutic efficacy and minimizing side effects. Finally, by using naturally sourced polysaccharides in hydrogel formulations, sustainability is promoted by reducing dependence on synthetic polymers, meeting the growing demand for eco-friendly materials. This review analyzes the interaction between marine polysaccharide hydrogels and encapsulating compounds and offers examples of how bioactive molecules can be encapsulated, released, and stabilized.

## 1. Introduction

Marine-derived polysaccharide hydrogels have emerged as a groundbreaking platform for the controlled release of natural bioactive compounds, offering innovative solutions across various fields, particularly in biomedicine. These hydrogels exploit the remarkable properties of polysaccharides, such as their biocompatibility, biodegradability, and water absorption capabilities, to create versatile matrices capable of effectively encapsulating and delivering bioactive substances. By incorporating marine-derived polysaccharides, which are inherently eco-friendly, sustainable, and abundant, these hydrogels present a promising alternative to synthetic polymers in various biomedical applications. Alginate, carrageenan, chitosan, agarose, and hyaluronic acid offer unique advantages in hydrogel design, particularly due to their specialized chemical structures and functional groups, making them attractive candidates for drug delivery, tissue engineering, and other therapeutic uses. One of the unique values of using marine sources for polysaccharides lies in the distinct chemical structures and functional groups these materials possess, which are crucial for creating efficient delivery systems. These polysaccharides have functional groups like sulfate, carboxyl, and hydroxyl groups that enable effective interactions with drugs, proteins, and other bioactive molecules, significantly improving loading efficiency and controlled release. These unique characteristics have the added benefit of creating gels that can respond to various environmental stimuli, such as pH, temperature, or ionic strength, which allows for more precise control over the release kinetics of bioactive compounds, and that can respond to the physiological conditions of the body, making them highly suitable for controlled release applications in drug delivery. Additionally, the natural gelation properties of marine-derived polysaccharides contribute to their swelling behavior, which is key for regulating the rate at which drugs or bioactive substances are released. This is particularly advantageous for developing hydrogels that can provide sustained and localized delivery of therapeutic agents. In contrast, polysaccharides derived from terrestrial sources often face significant limitations, including lower water solubility, poor stability in physiological conditions, and reduced mechanical strength compared to their marine counterparts. For instance, cellulose, although abundant and renewable, has a crystalline structure that makes it less responsive and more challenging to use in drug delivery systems. Similarly, polysaccharides such as starch and guar gum, while widely available, may lack the specialized functional groups that enable targeted interactions with drugs, cells, or other biomolecules, limiting their versatility in biomedical applications. Furthermore, many terrestrial polysaccharides exhibit lower biodegradability and can potentially lead to longer persistence in the body, raising concerns about toxicity or accumulation in the tissues [1]. Marine-derived polysaccharides offer several key benefits that address these limitations. Their inherent structural diversity, which includes sulfate and carboxyl groups, enhances their ability to interact with bioactive molecules, thus improving the encapsulation and release efficiency of therapeutic agents. The gelling properties of marine polysaccharides, such as those exhibited by agarose and carrageenan, further enhance their utility in drug delivery by forming stable three-dimensional networks that can encapsulate a wide range of compounds, from small-molecule drugs to proteins and nucleic acids. The gels can also be engineered to be responsive to specific environmental conditions, such as changes in pH, ionic strength, or temperature, providing an additional layer of control over the release profile of the encapsulated agents [1]. The chemical and physical properties of marine-derived polysaccharides underpin the numerous benefits they offer as materials for controlled drug delivery. From a chemical standpoint, the functional groups present in marine polysaccharides facilitate enhanced drug–polymer and polymer–polymer interactions, leading to improved drug loading, stability, and controlled release. These interactions are critical in creating hydrogels that not only effectively encapsulate bioactive compounds but also release them at therapeutic levels over extended periods. Additionally, the physical properties, such as swelling behavior and gelation mechanisms, further contribute to the controlled release by regulating the rate at which water or other solvents penetrate the hydrogel matrix, leading to gradual release over time. Another advantage of marine-derived polysaccharides is their contribution to the development of eco-friendly, sustainable biomaterials. Marine polysaccharides are typically biodegradable, meaning they break down naturally in the environment, reducing the risk of long-term accumulation and adverse effects. This biodegradability, combined with the natural origin of these materials, supports the development of environmentally conscious drug delivery systems that do not rely on synthetic, non-biodegradable polymers. This is especially important in the context of biomedical applications, where the long-term impact of materials on both human health and the environment is a critical consideration. This review comprehensively analyzes key marine-derived polysaccharides—namely alginate, carrageenan, chitosan, agarose, and hyaluronic acid—focusing on the fundamental mechanisms governing hydrogel formation and their physicochemical properties. Detailed attention will be given to recent advancements in utilizing these biopolymers as controlled delivery matrices for natural bioactive compounds and to the tailoring of hydrogel properties through variations in cross-linking strategies, molecular interactions, and hydrogel structure. Through an in-depth examination of the current literature, this review will show how marine polysaccharides can influence the release of encapsulated natural bioactives. Examples of biomedical applications will further illustrate the potential of these polysaccharide-based systems to serve as eco-friendly, sustainable carriers for bioactive substance delivery. Whether for drug delivery, wound healing, tissue engineering, or other therapeutic areas, marine-derived polysaccharide hydrogels offer considerable advantages over their terrestrial counterparts. Their ability to be chemically modified, their unique functional groups, and their environmental responsiveness make them promising candidates for the development of next-generation biomaterials, capable of improving patient outcomes and advancing medical technologies. By addressing both the therapeutic needs and sustainability goals, marine polysaccharide-based hydrogels present an exciting frontier in the development of innovative and effective biomedical products.

## 2. Polysaccharide-Based Hydrogels: Formation and Delivery Mechanism

**Figure 1:**
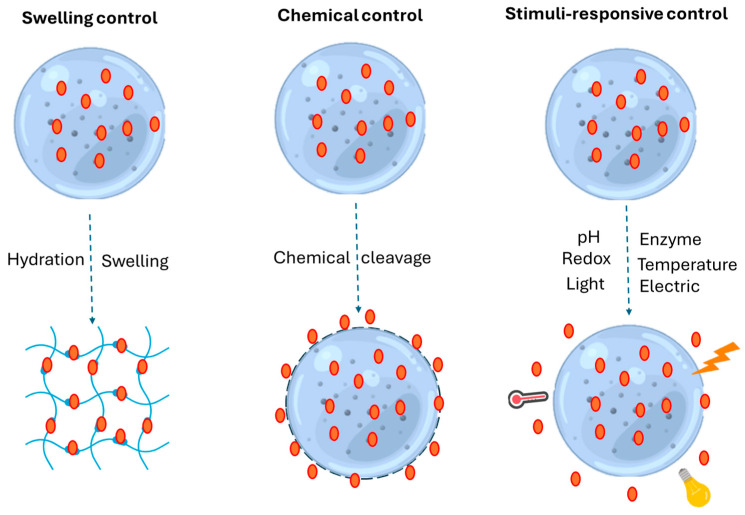
Release mechanisms of bioactive compounds from polysaccharide hydrogels.

Polysaccharide-based hydrogels derived from natural sources have garnered significant attention in recent years due to their biocompatibility, biodegradability, and versatility in various applications. These hydrogels are composed of long-chain carbohydrate polymers, such as alginate, chitosan, and hyaluronic acid, which can absorb large amounts of water while maintaining their structural integrity. The natural origin of these polysaccharides offers several advantages, including reduced toxicity, minimal environmental impact, and the ability to interact favorably with biological tissues [1]. As a result, they have found widespread use in fields such as biomedical engineering, drug delivery, tissue engineering, and wound healing [1]. Their unique ability to form hydrogels through physical or chemical cross-linking makes them adaptable for a wide range of functions, from acting as scaffolds for cell growth to delivering bioactive compounds in a controlled manner. The specific properties of hydrogels play a critical role in determining their suitability for various biomedical applications. For instance, their high water content allows hydrogels to mimic the extracellular matrix of natural tissues, creating a biocompatible environment that supports cellular activities and promotes tissue regeneration. Hydrogels can also be engineered to have tunable mechanical properties, making them suitable for applications ranging from soft tissues, such as cartilage or skin, to harder tissues, such as bone. The swelling behavior of hydrogels facilitates the controlled release of therapeutic agents, while their degradation kinetics can be adjusted to align with the desired treatment duration. Importantly, some hydrogels exhibit stimuli-responsive properties, such as sensitivity to pH, temperature, or specific enzymes, enabling precision in drug delivery and responsiveness to physiological changes. Furthermore, hydrogels provide protective encapsulation for biomolecules, shielding them from enzymatic degradation or immune recognition, thereby prolonging their stability and therapeutic efficacy. These properties collectively enable hydrogels to address the diverse and evolving demands of modern biomedical applications, from targeted drug delivery systems to scaffolds in tissue engineering [2]. However, achieving effective delivery of therapeutic biomolecules often necessitates a gentle hydrogel cross-linking process to maintain their integrity and activity. Cross-linking methods for polysaccharide-based hydrogels can be broadly categorized as physical or chemical [2]. Physical hydrogels are typically cross-linked through non-covalent bonds, which are reversible due to the weak interactions within the polysaccharide chains. These hydrogels can form without the need for cross-linking agents, enabling mild conditions for hydrogel formation, which is advantageous for preserving the structural and conformational integrity of biomolecules [3]. Physical cross-linking mechanisms include electrostatic interactions [4], hydrophobic interactions [5], ionic cross-linking with multivalent ions [6], van der Waals forces [7], and host–guest complexes [8]. Differently, chemically cross-linked networks are formed through irreversible bonds, often involving small multifunctional molecules like monomers, photo-reactive groups, or oligomers [9]. The development of marine-derived polysaccharide-based hydrogels for controlled drug delivery systems requires careful optimization of cross-linking strategies, molecular interactions, and hydrogel structure to enhance their effectiveness. Ionic cross-linking, commonly using divalent cations like calcium, offers simplicity and biocompatibility, though it can result in weak gels with low mechanical strength, limiting their use in certain applications. Covalent cross-linking, particularly via methods like photo-cross-linking, can improve stability and resistance to degradation but may introduce toxicity concerns if residual agents remain. Dual-cross-linking approaches, combining ionic and covalent methods, provide a balanced solution by enhancing gel strength and control over release properties. Molecular interactions, such as hydrogen bonding, electrostatic forces, and hydrophobic interactions, play a critical role in improving drug encapsulation and release profiles, but their complexity can make predicting release behavior challenging. Tailoring the hydrogel’s network density and incorporating controlled porosity can further optimize drug release, with denser networks slowing release and looser ones allowing for faster diffusion [2]. Moreover, with the optimization of hydrogel structures, marine-derived polysaccharide hydrogels can be further developed for effective, controlled drug delivery systems in biomedical applications. For example, functional groups within polysaccharide chains, such as carboxyl and amine groups, play crucial roles in covalent polymerization processes. Polysaccharides can be readily functionalized by incorporating reactive groups like thiols, alkenes, or acrylates, facilitating covalent cross-linking [10]. Both physically and chemically cross-linked hydrogels incorporate therapeutic biomolecules through weak interactions, adsorption within the structure, or cleavable bonds. Weak interactions provide tunable mechanisms for loading and releasing biomolecules, making polysaccharide-based hydrogels highly adaptable to various therapeutic needs. Through reversible physical adsorption or triggered chemical cleavage, these hydrogels ensure that therapeutic agents are released in a controlled, sustained, or responsive manner [11,12,13]. In particular, the integration of hydrogels into the delivery process produces a transformative shift in which hydrogels act as platforms that can profoundly alter the dynamics of biomolecule delivery due to their inherent characteristics. For example, their high water content mimics that of natural tissues, facilitating biomolecule diffusion and creating an environment conducive to cellular activities [14]. In addition, the properties of these hydrogels can be finely tuned to match specific requirements, including mechanical strength, swelling behavior, and degradation kinetics, ensuring optimal performance in biomolecule delivery [15] and in the capacity to accommodate a wide range of substances, from small molecules to proteins and nucleic acids [16]. Additionally, hydrogels can protect encapsulated biomolecules from degradation and immune recognition, prolonging their circulation time in the body [17]. Finally, some hydrogels display stimuli-responsive behavior, allowing for triggered release in response to external stimuli, adding an extra layer of control over biomolecule delivery dynamics [18,19,20]. Marine-derived polysaccharide hydrogels offer versatile mechanisms for the controlled release of therapeutic biomolecules. One mechanism is controlled delivery by diffusion and swelling, where the mesh size or porosity of the hydrogel network regulates biomolecule release [12], while matrix swelling allows water absorption without dissolution, enabling release during structural reorganization [21]. Another approach is stimuli-responsive controlled delivery, in which polysaccharides respond to environmental changes, such as ion concentration [22], light [23], pH [24], magnetic [25] or electric fields [26], temperature [27], redox potential [28], or specific biomolecules [29], facilitating on-demand release. Controlled delivery by degradation involves the natural biodegradation of the hydrogel, often accelerated by enzymes, to release entrapped biomolecules in a timed manner [30]. Affinity-based delivery exploits interactions between the biomolecules and the hydrogel matrix, allowing for tunable release based on affinity strength, ligand concentration, dissociation constant, and hydrogel properties [31]. Lastly, link-breaking-controlled delivery involves the covalent incorporation of biomolecules into the matrix, with release triggered by bond hydrolysis or other bond-breaking actions under specific physiological conditions, providing precise control over the release timing [32]. These diverse mechanisms highlight the potential of polysaccharide-based hydrogels as platforms for the controlled and responsive delivery of biomolecules (Figure 1).

## 3. Marine-Derived Polysaccharides

Marine resources represent a valuable source of natural materials for biomedical applications, providing both biological and economic benefits [33]. Many studies have demonstrated that polysaccharides derived from marine species can be effectively used for the delivery of biological molecules, such as antioxidant, anti-inflammatory, or anticancer agents, enhancing their stability and bioavailability in the biological system [34]. These materials can be utilized to formulate prolonged-release drugs because of their capacity to protect the active ingredient during transport through the body and release it gradually and selectively at the site of action [35]. These polymers can be used for the production of biomaterials, including hydrogels, scaffolds, and membranes, which have found applications in drug delivery, wound healing, and tissue engineering [36].

Marine-derived polysaccharide hydrogels, such as those made from alginate, carrageenan, agarose, chitosan, and hyaluronic acid, offer several advantages that make them attractive for biomedical use. Their natural origin ensures excellent biocompatibility and biodegradability, reducing the risk of toxicity and adverse immune reactions [16]. They can mimic the extracellular matrix of tissues due to their high water content and gel-like properties, creating a favorable environment for cellular activities such as adhesion, proliferation, and differentiation [16]. Additionally, these hydrogels are highly versatile, with tunable mechanical properties, swelling behavior, and degradation rates that can be adjusted to meet specific requirements [17]. This makes them suitable for a wide range of applications, from soft tissue scaffolds to controlled drug delivery systems. Furthermore, their ability to form hydrogels under mild conditions preserves the structural integrity and activity of encapsulated bioactive molecules. Some marine polysaccharides, such as alginate and chitosan, also possess inherent bioactivities, including antioxidant, antimicrobial, and anti-inflammatory properties, which can further enhance their therapeutic potential [17]. Another key advantage of marine-derived polysaccharide hydrogels is their adaptability to stimuli-responsive systems, enabling controlled drug release in response to changes in pH, temperature, ionic strength, or specific enzymes [27,28,29]. This responsiveness enhances the precision and efficacy of drug delivery, allowing for on-demand release at the target site. The abundance of functional groups in these polysaccharides also facilitates chemical modifications and the incorporation of bioactive ligands, broadening their utility in advanced biomedical applications. Despite these advantages, marine-derived polysaccharide hydrogels are not without limitations, which can hinder their broader application. One significant drawback is their relatively low mechanical strength, which can limit their use in load-bearing applications such as bone regeneration. This often necessitates blending with other polymers or reinforcing with nanoparticles to improve their structural integrity. Additionally, their degradation behavior can be unpredictable, with some hydrogels degrading too rapidly under physiological conditions, leading to a loss of functionality before the therapeutic objective is achieved. On the other hand, certain modifications required to control degradation rates can introduce chemical residues or alter the biocompatibility of the material [21]. Another challenge lies in the batch-to-batch variability of marine-derived polysaccharides, which arises from differences in species, geographical origins, and extraction methods. This variability can affect their physicochemical properties, making it difficult to ensure reproducibility and standardization in biomedical applications. Moreover, the extraction and purification processes for marine polysaccharides can be resource-intensive and environmentally taxing, potentially limiting their scalability and economic feasibility. Impurities introduced during these processes may also affect the performance and safety of the resulting hydrogels [21].

Additionally, while marine-derived polysaccharide hydrogels are generally biocompatible, their immunogenicity can vary depending on the specific polysaccharide and its processing. For instance, chitosan, derived from the exoskeletons of crustaceans, may carry allergenic risks for individuals sensitive to shellfish. Furthermore, some marine-derived hydrogels, such as those based on carrageenan, have been associated with inflammatory responses under certain conditions, necessitating further investigation into their safety profiles [20]. However, advances in material science, such as blending, chemical functionalization, and the development of hybrid systems, mitigated these issues and expanded their utility across a wider range of therapeutic and regenerative medicine applications.

### 3.1. Alginate

#### 3.1.1. Alginate: Origin and Properties

Alginate, a naturally occurring polysaccharide, has garnered significant interest due to its versatile properties. This polyanionic linear copolymer is primarily derived from brown algae of the Phaeophyceae family. It consists of β-D-mannuronic acid (M-block) and α-L-guluronic acid (G-block) residues, linked by β-1,4-glycosidic bonds [37]. The specific composition and sequence of these residues can vary depending on the source and species used for alginate extraction [37]. The mechanical properties of alginate are influenced by the length of the G-block units, while the M-block content affects its immunogenicity and viscosity [37]. Alginate is a hydrophilic and water-soluble substance that gels in mild circumstances with non-toxic reagents or with a variety of chemical or physical cross-linking techniques. Its natural abundance, biocompatibility, ease of handling, non-toxicity, and low cost make alginate a valuable polymer. Key properties such as porosity, swelling behavior, biodegradability, gel strength, and immunological compatibility are significantly influenced by composition, molecular weight, and the presence of cations [38]. Indeed, cations such as calcium play a crucial role in gel morphology by either promoting or inhibiting intermolecular interactions. For instance, calcium ions can induce the formation of a more robust network, while other cations might lead to a more porous and unstable gel structure. The kinetics of gelation is also critical in controlling the gelation process, with slower gelation rates ensuring the mechanical integrity of homogeneous gel structures [39].

#### 3.1.2. Alginate-Based Hydrogel Applications

**Figure 2:**
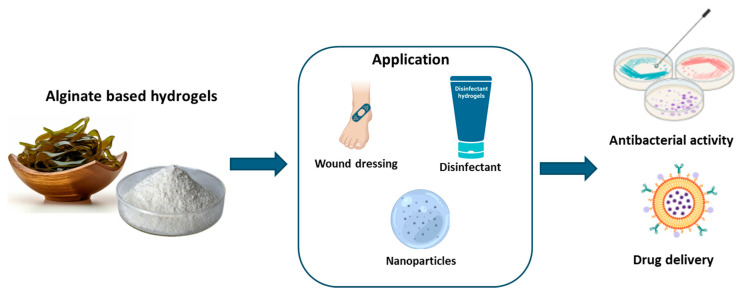
Aliginate-based hydrogel application.

Alginate produces a complex hydrogel with divalent ions, particularly calcium. This property is exploited to capture bioactive compounds, resulting in microencapsulation technology. Microencapsulation with alginate includes the development of small microspheres carrying the desired active component. These microspheres can be utilized to control the release of bioactive molecules like medicines, vitamins, and probiotics [40]. Alginate can produce hydrogels by replacing the sodium ions in glucuronic acids with multivalent cations, which bond the polymer chains [41]. Nevertheless, the hydrogel produced with this approach frequently has limited mechanical characteristics and thermal stability, and it can disintegrate under physiological environments. As a result, numerous researchers are investigating alternative cross-linking technologies, including chemical cross-linking and interpenetrating network technology [42]. Alginate, due to its nature and specific functional groups, can be easily combined with other biopolymers such as collagen, hyaluronic acid, agarose, and chitosan in order to produce hydrogels that are suitable for use as wound healing biomaterials [43]. Alginate-based products are very useful in the field of antibacterial materials due to their capacity to absorb liquids, keeping the wound surface dry [44]. Alginate-based hydrogels have been widely utilized for the delivery of natural substances, demonstrating their versatility and effectiveness in various applications. For example, it was demonstrated that dual-cross-linked (ionically and covalently) sodium alginate hydrogel coupled with varying quantities of honey (2% to 10%), using calcium cations and maleic anhydride, has consistent swelling properties and limited degradation of the polymer which results in increased healing efficacy and promotion of cell growth and proliferation. Experiments on in vivo wound contraction kinetics applied on murine models and monitored by both histopathology and “Swept Source Optical Coherence Tomography imaging” evidenced improved wound closure and higher epithelial thickness. Moreover, this wound dressing showed strong antibacterial activity against methicillin-resistant strains of *S. aureus* and *E. coli* [45]. Likewise, another study found that combining alginate and chitosan with aloe vera extract and honey can produce effective and biocompatible hydrogels for the healing of injured skin lesions. Each component utilized helped to improve the physical and structural features of the hydrogels, making them perfect for stimulating cell adhesion, proliferation, and migration [43]. Furthermore, the efficacy of the components in preventing bacterial infections from Staphylococcus aureus and Pseudomonas aeruginosa was underlined. The results showed that the porous structure with interconnected cavities of the hydrogel provides suitable conditions for cell adhesion, migration, and proliferation and their structural properties can be a strong suit to wound healing. Although honey’s application can weaken the hydrogel structure, the addition of aloe vera improved the hydrogel’s specificity for wound healing [46]. In a study by Rusu et al. [47], the controlled release capacity of Lavender Essential Oil (LVO) from gel formulations was assessed utilizing in vitro assays. The hydrogels under examination were created by combining Poly[itaconic-anhydride-co-3,9-divinyl-2,4,8,10-tetraoxaspiro(5,5)]undecane with sodium alginate, cross-linking it with Phytic Acid, and adding LVO to impart antibacterial and antioxidant features. The inclusion of LVO increased all formulations’ antibacterial effectiveness against S. aureus and C. albicans while retaining biocompatibility [47]. Clemente et al. [48] conducted a study that used two biocompatible and biodegradable polymers, sodium alginate and sodium carboxymethyl cellulose, to generate cross-linked hydrogel beads. These beads were utilized to encapsulate and release glycoalkaloids derived from tomato and potato leaves in a regulated manner, allowing them to be employed as biocompatible disinfectants [48]. Different studies were undertaken on the application of alginate-based hydrogels for the regulated or sustained release of various bioactive compounds, including quercetin [49] and curcumin [50]. Quercetin-loaded alginate beads, prepared by ionic gelation, have been shown to possess a microstructure characterized by homogeneously distributed polymer aggregates and precipitated quercetin within the particle matrix, which can potentially enhance drug loading capacity. The swelling and erosion phenomena of these beads, influenced by surface imperfections such as cracks and holes, play a crucial role in controlling the non-homogeneous release of quercetin [49]. Similarly, curcumin encapsulated in alginate hydrogel matrices has demonstrated significant potential in managing cancer and chronic wounds. Despite curcumin’s limitations, including poor bioavailability, hydrophobicity, and rapid systemic clearance, alginate hydrogels resulted in being a promising platform to overcome these challenges, emphasizing the mechanistic effects of curcumin inside cells and the parameters regulating its release behavior [50]. Table 1 and Figure 2 recap the applications of alginate-based hydrogels.

### 3.2. Carrageenan

#### 3.2.1. Carrageenan: Origin and Properties

Carrageenan, derived from the red algae Rhodophyceae, is a high-molecular-weight linear polysaccharide formed by galactose and 3,6-anhydrogalactose residues linked alternatively by α-1,3 and β-1,4 glycosidic bonds [51]. This polymer dissolves in water to generate very viscous solutions. The viscosity differs on the concentration, temperature, kind of carrageenan, and molecular weight [52]. Carrageenan has been demonstrated to possess unique pharmacological qualities, such as antihyperlipidemic, anticoagulant, anticancer, and immunomodulatory activities. Furthermore, it has been shown to be as effective as anti-inflammatory, antiviral, and antioxidant agents [53]. In addition, carrageenan has been shown to have potent antibacterial properties against Gram-positive bacteria, including Listeria monocytogenes [54].

#### 3.2.2. Carrageenan-Based Hydrogel Applications

**Figure 3:**
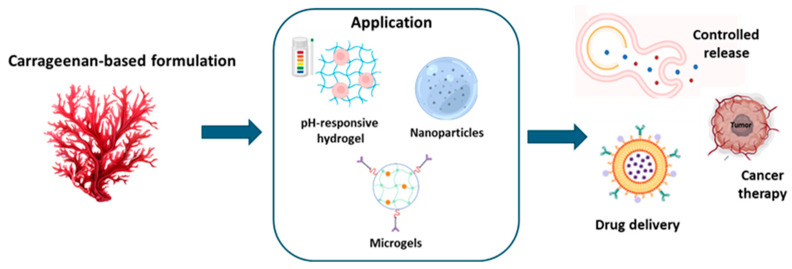
Application of carrageenan-based formulation.

Since it is known for its rheological features, carrageenan is frequently utilized in hydrogel production. The resultant gels are ionically and thermally reversible. Carrageenan cations, such as potassium ions, play an important role in the development of the gel. Moreover, the addition of sugars and polyols enhances gel formation capabilities [55,56]. Carrageenan-based gels are brittle and mechanically unstable under physiological settings due to their high swelling ratio [57]. However, this constraint can be avoided by chemically modifying the polymer structure, while also considering the abundance of functional groups capable of improving the physicochemical properties of the hydrogel. Indeed, the polymer can undergo a variety of modifications, including oxidation [58], oversulfation, acetylation, phosphorylation [59], carboxymethylation [60], and methacrylation [61]. These adjustments not only increase the strength of the polymer but also give the hydrogel new functionalities and specific properties such as enhanced porosity. For example, Varghese et al. [62] evaluated the influence of porosity and pore distribution on the release of quercetin. By raising the carrageenan ratio, quercetin release increased, revealing a direct relationship between the porosity of the hydrogel and the amount of quercetin released. Carrageenan is also widely employed as a gelling, stabilizing, and thickening agent in pharmaceutical and industrial fields [63] and is used as a vehicle for the regulated delivery of drugs and bioactive compounds [64,65]. In fact, alginate/carrageenan microgels improve immunoglobulin retention, loading efficiency, and release amount. For instance, this system has been developed for encapsulating and releasing egg yolk immunoglobulin Y (IgY) under simulated gastrointestinal conditions. The addition of κ-carrageenan, particularly at a concentration of 0.30%, optimized the encapsulation and loading efficiency of IgY and enhanced its stability. Compared to pure alginate beads, composite beads demonstrated superior performance, showing no loss in IgY activity after exposure to simulated gastric fluids, while pure alginate beads exhibited around 35% loss. This enhanced stability was attributed to a stronger electrostatic attraction and a more densely packed biopolymer network within the composite beads, as evidenced by their electrical and microstructural properties. Moreover, the composite beads displayed resistance to swelling in the stomach phase, effectively protecting the encapsulated IgY, while allowing controlled swelling and release in the intestine phase [66]. Additionally, carrageenan/Pectin gels protect β-galactosidase by decreasing enzyme sensitivity and increasing compound stability during digestion. Indeed, these hybrid hydrogels, synthesized using the ionotropic gelation method, were characterized through Fourier transform infrared spectroscopy (FTIR), thermogravimetric analysis (TG/DTG), and scanning electron microscopy (SEM). Encapsulation efficiency was found to be 77 ± 2%. Moreover, carrageenan increased stability and was 2.0 times more effective than commercial tablets in releasing β-galactosidase [67]. pH-sensitive hydrogels are currently being studied as carriers for the controlled release of drugs in a pH-specific environment [68,69]. Xie et al. [70] investigated the retention rate of blueberry anthocyanins at different pH levels (1.0–6.0) and developed a new hydrogel composed of κ-carrageenan-containing nanocomplexes able to encapsulate blueberry anthocyanins. The results showed that the hydrogel successfully safeguards against anthocyanin degradation at low pH, with a considerably better retention rate at pH 6.0 than at pH 1.0 [70]. When developing carrageenan-based delivery systems, the type and concentration of carrageenan affect vehicle formation. In a study on whey protein/κ-carrageenan hydrogels for colon-specific curcumin administration, κ-carrageenan was found to preserve curcumin from degradation during digestion. Hydrogels with 0.55% κ-carrageenan showed lower curcumin degradation compared to those with 0.1% κ-carrageenan. κ-carrageenan protects proteins during in vitro digestion, decreasing the activity of digestive enzymes on hydrogels and preventing the degradation of the gel matrix and the release of curcumin [71]. In a study by Russo et al. [72], curcumin was used as a pharmacological model to evaluate the action of beads made of cross-linked carrageenan and alginate. Poloxamer 407, a non-ionic surfactant that can also create hydrogels, was added to the beads’ composition to boost the solubility of curcumin. Such composite hydrogels have enhanced drug solubility, metabolic stability, and non-toxicity, as well as the capacity to target cancer cells more specifically [72]. Table 2 and Figure 3 recap the applications of carrageenan-based hydrogels.

### 3.3. Chitosan

#### 3.3.1. Chitosan: Origin and Properties

Chitosan is a naturally occurring polymer consisting of β-(1→4)-linked D-glucosamine and N-acetyl-D-glucosamine with a pKa value varying from 6.3 to 6.5. It is a cationic and muco-adhesive polysaccharide obtained by the deacetylation of chitin mostly extracted from crustacean shells, but also from insect exoskeletons and some fungi and algae [73]. The increase in chitosan deacetylation proportionally enhances its biocompatibility and biodegradability. The charge density of this polysaccharide depends on the degree of deacetylation due to the amino group, and the pH of chitosan in solution depends on the quantity of ionized amino groups [74]. Chitosan assumes great importance due to its characteristics of biocompatibility, degradability, and antibacterial properties [75]. Even if the structural characteristics indicate limited chain flexibility and poor mechanical strength of chitosan, due to the abundance of hydroxyl and amine groups on the polysaccharide chain, cross-link interactions can be created, allowing for the production of flexible three-dimensional hydrogel structures [76].

#### 3.3.2. Chitosan-Based Hydrogel Applications

**Figure 4:**
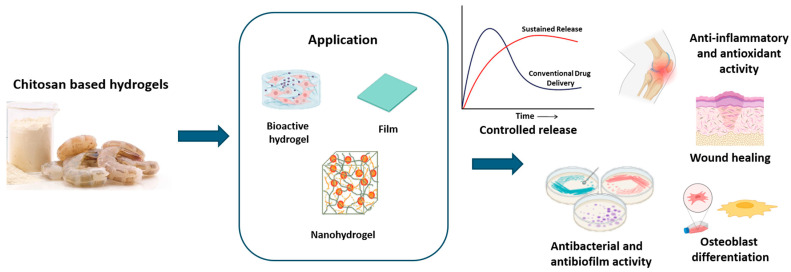
Chitosan-based hydrogel application.

The cationic character of chitosan, along with the different types of non-covalent interactions (such as hydrogen bonds and hydrophobic and electrostatic interactions), covalent bonds (such as imine and disulfide bonds) [77], and a combination of covalent and physical cross-linking that can be used to create performing hydrogels, make this biopolymer highly performing in producing hydrogels for drug delivery [78]. The complex network formed in chitosan-based hydrogels allowed the incorporation of several bioactive compounds in a versatile manner [79]. Furthermore, chitosan-based hydrogels prove to be an alternative to conventional biodegradable materials thanks to their cost-effectiveness, low toxicity, and environmentally friendly degradation [80]. Bioactive compounds, such as proteins, amino acids, polyphenols, nucleic acids, drugs, and natural extracts can be encapsulated in chitosan-based hydrogels to create appropriate delivery systems. A recent study reported the preparation of chitosan hydrogels in whose microstructure phloroglucinol, a natural phenolic compound, has been incorporated [81]. The natural phenol adsorbed on chitosan made the structural network more compact, as well as enhanced its antioxidant (up to 25%) and antimicrobial activities. These characteristics make such hydrogels particularly adaptable for the formulation of dressings for wound healing, thanks to their hemocompatibility and the capacity to increase the proliferation of fibroblasts [81]. A chitosan/silk hydrogel loaded with purified polysaccharides from Curcuma zedoaria and platelet-rich plasma–exosomes was synthesized by Xu N. et al. [82]. This device showed a wound contraction in diabetic rats and the combination of polysaccharides and exosomes enhanced the activity [82]. Chitosan-based gels containing vitexin, a glycosylated flavone plant extract with antioxidant, anti-inflammatory, antiviral, and antibacterial properties, have been used for the treatment of excisional wounds [83]. Tests on HaCaT cells demonstrated significant cell migration after 72 h and, in general, a decrease in wound diameter was observed, compared to the control [83]. Chitosan hydrogels incorporating Fagonia indica improved skin wound re-epithelialization and sped up wound healing in vivo [84]. This gel showed antibacterial properties against Pseudomonas aeruginosa, due to the combined effect of high flavonoid content of the extract and the chitosan. Similarly, the polyphenolic extract from Hamamelis virginiana, oxidized by laccase and cross-linked with chitosan/gelatin, allowed for the creation of a functionalized platform for wound reparation [85]. This hydrogel was able to release the phenolic compounds inhibiting metalloproteases, reactive oxygen species, showing antibacterial activity against P. aeruginosa and S. aureus [85]. Chitosan-based hydrogels loaded with Punica granatum [86], Periplaneta americana extract [87], aloe vera juice [88] and gel [89], yellow tea [90], Bletilla striata [91], Salvia officinalis [92], and Salix alba [93] represent a versatile and promising class of biomaterials for wound care due to their multifaceted properties. These hydrogels combine regenerative effects on skin wounds with anti-inflammatory and antibacterial activities, addressing key challenges in wound healing, such as infection control and tissue regeneration. Their ability to release bioactive compounds at a slow, controlled rate ensures prolonged therapeutic efficacy while reducing the need for frequent reapplications, which is particularly beneficial for chronic or complex wounds. The incorporation of Periplaneta americana extract into hydrogel films has shown remarkable wound healing potential by accelerating re-epithelialization, enhancing collagen deposition, and promoting angiogenesis. These effects are achieved through the activation of the TGF-β/Smad signaling pathway, which plays a critical role in tissue repair and regeneration. The hydrogels demonstrated excellent swelling capacity and water vapor transmission rates, making them ideal for wound dressing applications where moisture balance is crucial [87]. Chitosan-based hydrogels loaded with aloe vera juice exhibited improved tensile strength, enhanced elongation properties, and reduced surface roughness, contributing to their mechanical stability and biocompatibility. These properties make them highly effective in maintaining a moist wound environment while providing antimicrobial protection against common pathogens. Additionally, these hydrogels displayed non-cytotoxicity toward murine fibroblasts, ensuring their safety for clinical applications [88]. The inclusion of yellow tea extract in hydrogel matrices significantly improved their swelling ability and interaction with physiological liquids. The hydrogels exhibited a reduction in contact angles by up to 60% during incubation, indicating enhanced wettability and potential for improved bioavailability of active compounds. Despite these structural changes, the hydrogels maintained their bioactivity, making them a viable option for advanced wound healing formulations [90]. Bletilla striata-based hydrogels, when combined with carboxymethyl chitosan (CMC) and carbomers, demonstrated optimal characteristics such as a porous structure, elastic properties, and superior water retention. These hydrogels promoted wound closure rates of over 83% within 14 days, supported by histological evidence of re-epithelialization, dense collagen fiber deposition, and neovascular formation. Their hydroxyl radical scavenging properties further enhanced their therapeutic potential by reducing oxidative stress at the wound site [91]. Hydrogels enriched with Salvia officinalis [92] and Salix alba [93] extracts demonstrated potent antibacterial effects against a range of standard laboratory strains, including *Staphylococcus aureus* and *Pseudomonas aeruginosa*. These hydrogels were characterized by their three-dimensional cross-linked structures, providing enhanced stability and bioactive compound retention. The hydrogels showed no cytotoxic effects on human embryonic kidney (HEK-293) cells and demonstrated hemocompatibility, ensuring their safety and effectiveness as wound dressings [92,93]. Moreover, hydrogels with Punica granatum extract exhibited high antioxidant capacity, further aiding the wound healing process by neutralizing free radicals and reducing inflammation. This antioxidant activity, combined with the intrinsic biocompatibility of chitosan, positions these hydrogels as a robust platform for the development of next-generation wound care products [86]. Lawsonia inermis ethanolic extract enriched in terpenoids, alkaloids, tannins, and saponins was loaded in chitosan/PVA hydrogels [94] with a slow release of about 50% of the drugs, highlighting interesting wound healing properties. Essential oils from copaiba rich in β-caryophyllene [95], along with those produced from Eucalyptus, Ginger, and Cumin that contain tannins, phenols, and terpenoids [96,97], were loaded in chitosan-based hydrogels, highlighting an acceleration in wound healing and optimal antibacterial and anti-inflammatory effects. Bee honey, rich in phenols and enzymes, was incorporated into a chitosan-based hydrogel allowing for the maintenance of a well-structured epidermis and accelerating wound healing [98]. A chitosan-coated semi-interpenetrating polymer hydrogel, containing sodium alginate and poly(2-ethyl-2-oxazoline), was loaded with Thymol (2-isopropyl-5-methylphenol) derived from plants like Thymus vulgaris, Ocimum gratissimum, and Satureja thymbra [99]. The monoterpene phenol used in chitosan hydrogels was able to stimulate osteoblastic differentiation in mouse mesenchymal stem cells, promoting its application in bone strengthening [99]. For the administration of natural products such as quercetin, calcium carbonate microcapsules containing nanohydroxyapatite/chitosan/collagen hydrogel particles were characterized by prolonged-release profiles of flavonoids, which has the property of treating bone diseases by promoting their regeneration [100]. Furthermore, phenethylisothiocyanate, a bioactive phytochemical from cruciferous vegetables, was loaded in an injectable chitosan/pluronic hydrogel to improve its water solubility and half-life. The hydrogel was able to reduce joint edema and the advancement of arthritis and bone erosion [101]. Zhang et al. [102] produced in situ gelling chitosan hydrogels able to locally release cannabidiol, for the treatment of spinal cord lesions. They found a prolongation of administration for 72 h and a reduction in spinal cord lesions [102]. Chitosan-based injectable hydrogels were also used due to their characteristic of becoming gelling matrices at the injection site [103]. For example, chitosan hydrogels formulated for the sustained release of caffeic acid phenyl esters have been tested for the treatment of periodontitis showing an effective reduction in inflammation and a simultaneous repair of bone tissue [104]. Chitosan hydrogels encapsulating curcumin showed inhibitory effects against Streptococcus mutants, representing a potentially effective remedy against dental decay-fighting [105]. Similarly, turmeric cyclodextrin-grafted chitosan hydrogels were studied by Hao et al. [106]. They found antimicrobial activity against Staphylococcus aureus and E. coli [106]. Chitosan nanohydrogels loaded with tanshinone, a type of diterpenoid from Salvia miltiorrhiza, were formulated to obtain antibacterial and anti-biofilm activity against Streptococcus [107]. Tanshinone was characterized by a synergistic antibacterial/anti-biofilm effect under acidic conditions [107]. Zhou et al. [108] explored the possibility of creating a rhein (4,5-dihydroxyanthraquinone-2-carboxylic acid)−chitosan hydrogel with mechanical strength, sustained release, and low toxicity characteristics. Rhein, an anthraquinone compound mainly isolated from the herbal medicine rhubarb, was used as an antineuroinflammatory agent [108]. Other bioactive compounds with anti-neuroinflammatory activity from Aster glehni leaves were also used to improve chitosan hydrogels for neuritis treatment [109]. Promising encapsulating methods for Yerba mate with chitosan nanoparticles have been utilized as vehicles for caffeoyl derivatives and other phenolic compounds [110]. Chitosan hydrogels were also used for the incorporation of tea tree oil for acne treatment. The antimicrobial activity of the hydrogel was improved compared with the essential oil alone, allowing for a reduced dose of the oil [111]. Plant extracts such as carvacrol (phenol monoterpene) [112] or quercetin [113] were encapsulated into chitosan hydrogels and used as a pH-sensitive delivery platform for anticancer purposes. The same pharmacological activity was obtained by encapsulating resveratrol into chitosan-based hydrogels, obtaining a rapid absorption into simulated gastric fluid [114], and with chitosan-coated alginate microbeads containing leaf extracts of Moringa oleifera, which exhibited an improved stability [115]. Table 3 and Figure 4 recap the described systems.

### 3.4. Agarose

#### 3.4.1. Agarose: Origin and Properties

Agarose (AGR) is a water-soluble natural polysaccharide derived from red marine algae (Rhodophyceae). It is made up of repeated agarobiose units coupled in a linear form with bonds (1–3) of β-D-galactopyranose (1–4) and linked to 3,6-anhydro-α-L-galactopyranose [116,117]. The rigidity and functional groups of agarose enable it to stimulate cell adhesion, proliferation, and activity, making it an ideal substrate for the culture of microorganisms such as bacteria and yeasts. This feature makes it suitable for the production of biomass and bioproducts. Furthermore, the water absorption capacity of agarose can be adjusted, providing cells with the ideal microenvironment for their activity and making agarose especially appealing for numerous biomedical applications [118,119]. Agarose can be mixed with other polysaccharides and peptides to produce more complex structures with improved physical properties. For example, mixing agarose with low gel point thermosensitive biopolymers can produce stable viscoelastic gels with specific barrier properties dictated by the type and concentration of the biomacromolecules incorporated into the resulting hybrid [120].

#### 3.4.2. Agarose-Based Hydrogel Applications

**Figure 5:**
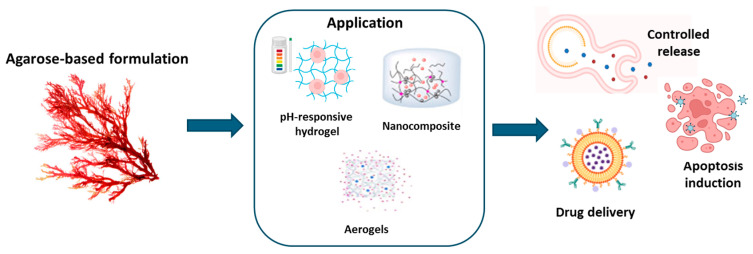
Application of agarose-based formulation.

Agarose is widely employed in the production of particles, hydrogels, microcapsules, and microspheres. Indeed, agarose hydrogels are utilized to build drug delivery systems based on the biocompatibility and hemocompatibility properties of this material [121]. Moreover, agarose hydrogels are electrically conductive, pH-sensitive, and thermally reversible, which makes them ideal for the loading and control of drug release in response to various environmental conditions, extending the drug’s residence time in the body and lowering the need for frequent dosing as well as the adverse effects of the drug [122]. Furthermore, the ability to change agarose gelation properties through chemical modifications allows for the creation of hydrogels with varying mechanical and bioactive properties for a wide range of applications. Researchers have been successful in producing agarose gels with low gelling temperatures using acetylation, alkylation, alkenylation, acylation, and oxyalkylation [123,124] or in creating innovative materials, containing unique functional groups such as tosyl or amino moieties [125]. This variety of agarose modification procedures allows for the tuning of hydrogel features such as cross-link density, porosity, and active substance release capacity [126]. Agarose is frequently used in biomedical applications such as cell treatment, tissue engineering, and targeted drug delivery of natural substances. The diffusion characteristics of natural substances from AGR gels are closely related to the rheological properties of the gels. AGR derivatives with low gelation temperatures enable faster substance delivery because the double helicoidal structure, which forms during gelation, is reduced, resulting in a lowered storage modulus [127]. Ninan N et al. [128] demonstrated that a pH-sensitive carboxylated agarose hydrogel mixed with zinc ions and tannic acid had an excellent wound healing function by boosting cell migration and proliferation, all of which are essential for wound healing. This product’s capacity to adjust to variations in the pH of the skin makes it ideal for usage in a variety of clinical settings, assuring consistent and targeted action [128]. Agarose gels are capable of swelling within an aqueous solution, allowing for drug loading by immersing the agarose in the drug solution and collecting the inflated hydrogel once maximum drug diffusion has occurred [129]. The variation in drug concentration between the water-based medium and the gel structure works as a driving force, causing drug molecules to diffuse into the porous structure. In the absence of contacts, drug release is primarily mediated by mass diffusion, also known as Fickian diffusion, the time of which is determined by the network topology and particle size [129]. This behavior was used by Rajabzadeh-Khosroshahi et al. [130] to develop a degradable and biocompatible nanocomposite that contains chitosan, agarose, and graphitic carbon nitride to load and release anticancer curcumin. The results showed that the nanocomposite particles improved the bioavailability of curcumin in pH-sensitive drug release experiments [130]. Similar results were obtained with flexible pH-responsive agarose/succinoglycan hydrogels. These hydrogels have significantly improved flexibility, thermostability, and porosity compared to agarose gels alone. The mechanical and physical properties of agarose/succinoglycan hydrogels were extensively investigated using various instrumental techniques, including rheological measurements, attenuated total reflection–Fourier transform infrared spectroscopy (ATR-FTIR), X-ray diffraction (XRD), and field-emission scanning electron microscopy (FE-SEM). The findings revealed that the agarose/succinoglycan hydrogels formed flexible and stable network gels with an enhanced swelling pattern in basic solutions, as opposed to the hard and brittle nature of agarose gels alone. Furthermore, these hydrogels exhibited a pH-responsive release profile for drugs, achieving a cumulative release of approximately 41% within 35 h at pH 1.2 and a complete release at pH 7.4 [131]. Samadi and coworkers [132] included quercetin in a pH-responsive hydrogel nanocomposite made from agarose, polyvinylpyrrolidone, and hydroxyapatite. Quercetin was encapsulated in the internal aqueous phase of a water/organic/water emulsion with improved loading capacity, sustained release, and apoptosis-inducing actions. In particular, the pH-responsive release of quercetin had sustained release over a period of 96 h. According to the Korsmeyer–Peppas mathematical model, the release mechanism was diffusion-controlled at pH 7.4 and dissolution-controlled at pH 5.4. The presence of all components was confirmed by Fourier transform infrared analysis, while X-ray diffraction results validated the incorporation of quercetin within the nanocomposite. Field-emission scanning electron microscopy images showed a homogeneous surface of the nanocomposite, indicating good compatibility between all components. Zeta potential analysis confirmed the stability of the nanocarriers. Furthermore, this platform demonstrated significant cytotoxicity on MCF-7 cells when compared to free quercetin, with enhanced apoptosis induction [132]. Table 4 and Figure 5 recap the described systems.

### 3.5. Marine Hyaluronan

#### 3.5.1. Hyaluronan: Origin and Properties

Hyaluronic acid (HA) is a linear anionic polysaccharide belonging to the glycosaminoglycan family, composed of alternating units of N-acetyl-D-glucosamine and glucuronic acid [133]. Traditionally, HA is extracted from rooster combs and bovine vitreous humor, but it is also commonly derived from marine sources, including fisheyes, sharks (skin and cartilage), swordfish, zebrafish, mollusk bivalve, liver of stingrays, and shellfish [134]. In humans, HA is present in synovial fluid and the vitreous humor of the eye, playing a crucial structural role in articular cartilage and skin [135]. HA is water-soluble and forms highly viscous solutions, exhibiting free radical scavenging properties, promoting bacteriostasis, and aiding in tissue repair [136]. However, the HA homopolymer lacks sufficient strength and fluidity to serve as a supportive scaffold on its own. To address this, HA is cross-linked with ethyl esters, benzyl esters, or other biodegradable polymers to improve its mechanical properties while maintaining biocompatibility [137]. These cross-linked HA hydrogels are highly versatile, capable of being fabricated into sheets, membranes, sponges, tubes, fibers, and scaffolds for applications such as wound healing, tracheal regeneration, and the repair of cartilage, vasculature, and nerve tissues [137].

#### 3.5.2. Hyaluronan-Based Hydrogel Applications

**Figure 6:**
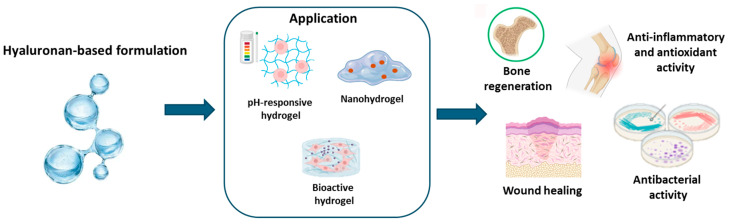
Application of hyaluronan-based formulation.

HA-based hydrogels have gained significant attention in biomedical fields such as drug delivery, tissue engineering, and regenerative medicine due to their biocompatibility, biodegradability, non-immunogenicity, responsiveness to environmental cues, and adjustable properties [138,139,140]. For example, Kwon et al. synthesized pH-sensitive hydrogels containing isoliquiritigenin, a phenolic chemical compound found in licorice, using hydroxyethyl cellulose and HA for transdermal drug delivery. The hydrogels, formed via Michael addition, demonstrated pH-dependent drug release, with higher drug release at pH levels above 7 [141]. A composite hydrogel incorporating tannic acid and dopamine-coated carbon particles rich in phenolics has been developed by Xin Jin et al. to enhance cross-linking sites through oxidative coupling and improve adhesion via covalent and hydrogen bond formation between the hydrogel and wound tissues [142]. This hydrogel featured a rapid gelation time (<6 s) and exceptional adhesive strength (>8.1 kPa). In vitro studies confirmed the hydrogel’s low hemolytic activity, minimal cytotoxicity, and ability to promote fibroblast proliferation and migration. In vivo experiments using a full-thickness skin defect model showed that the hydrogel accelerates wound healing under mild photothermal stimulation from the DCPs by reducing inflammation, alleviating tissue hypoxia, and promoting angiogenesis and epithelialization [142]. Gallelli et al. [143] evaluated the clinical efficacy and the safety of HA-based nanohydrogels embedded with quercetin and oleic acid in the treatment of lower limb skin wounds in patients with diabetes mellitus. The treatment with HA hydrogels reduced the wound healing time, in comparison to free hyaluronic acid (0.2%), without developing adverse drug reactions, suggesting that this formulation could be used in the management of wound healing [143]. Similarly, Conte et al. [144] used the extract of Opuntia ficus-indica, known for its anti-inflammatory, antioxidant, and antimicrobial properties, encapsulated in chitosan nanoparticles embedded in pluronic–hyaluronic thermo-responsive hydrogels to achieve localized and sustained release in periodontal pockets. This system eradicated biofilms of S. mutans, P. aeruginosa, and P. gingivalis and disrupted extracellular polymeric substance formation. Moreover, such gels modulate immune responses [144]. The same authors reported the preparation of hyaluronic acid hydrogels containing resveratrol-loaded chitosan (CS) nanoparticles for the treatment of atopic dermatitis. Embedding the nanoparticles in HA delayed their hydrolytic degradation and slowed resveratrol release. This system decreased oxidative damage in TNF-α/INF-γ-treated human keratinocytes (HaCaTs) and reduced the secretion and gene expression of proinflammatory cytokines [19]. Valentino et al. [20] developed a localized drug delivery platform containing Hydroxytyrosol-loaded chitosan nanoparticles into an in situ hydrogel composed of pluronic F-127 and hyaluronic acid. Such a system can be injected into a target region as a flowing solution, forming a gel at body temperature. This hydrogel reduced oxidative and inflammatory effects in the chondrocyte cellular model and influenced chondrocyte gene expression under a pathological state [20]. The described examples are summarized in Table 5 and Figure 6.

## 4. Conclusions

Marine-derived polysaccharide hydrogels represent an exciting advancement in material science due to their wide range of applications and inherent advantages. They are particularly valuable in the biomedical field, where they are used in wound dressings and as drug delivery systems. Their high water content and biocompatibility make them excellent mimics of natural tissue, creating an environment that promotes cell growth, tissue repair, and healing. Moreover, their ability to protect bioactive compounds from premature degradation while ensuring gradual and targeted release enhances the effectiveness of therapeutic treatments. In drug delivery, the customization of polysaccharide-based hydrogels offers the potential to address specific medical needs, including the treatment of chronic diseases and localized infections. By modulating factors such as cross-linking density and responsiveness to pH, temperature, or enzymes, these hydrogels can be engineered to release drugs in a controlled manner, improving bioavailability and minimizing side effects. For instance, in cancer therapy, polysaccharide-based hydrogels can be designed to release anticancer agents at the tumor site while sparing healthy tissues, offering a safer and more effective treatment option. As research into marine-derived polysaccharide hydrogels continues, advancements in their design and functionality are expected to push the boundaries of their applications. For instance, the development of multi-responsive hydrogels that can react to multiple environmental cues simultaneously could open up new possibilities for smart drug delivery systems and self-healing materials. Furthermore, with the rising interest in sustainable materials, marine-derived polysaccharide hydrogels align with global trends towards reducing the environmental impact of processes. By harnessing natural, renewable polysaccharides from marine sources, these hydrogels contribute to the circular economy, lowering the dependence on non-biodegradable and petroleum-based materials. In conclusion, marine-derived polysaccharide hydrogels are a promising platform not only for the controlled release of natural bioactive compounds but also for a broad spectrum of applications in medicine. Their versatility, coupled with their eco-friendly and biocompatible nature, ensures that they will remain at the forefront of material science innovations in the years to come.

## Figures and Tables

**Table 1:** Applications of alginate-based hydrogels.

Application	Alginate-Based Formulation	Benefits	References
Wound dressing	Alginate hydrogel with honey	Strong antibacterial activity against methicillin-resistant strains of S. aureus and E. coli	[45]
Wound dressing	Alginate and chitosan with aloe vera extract and honey	Prevention of bacterial infections from Staphylococcus aureus and Pseudomonas aeruginosa	[46]
Multifunction hydrogel	Lavender Essential Oil in alginate-modified hydrogel	Antibacterial effectiveness against S. aureus and C. albicans	[47]
Biocompatible disinfectants	Cross-linked hydrogel beads encapsulating glycoalkaloids derived from tomato and potato leaves	Antibacterial properties	[48]
Multifunction hydrogel	Alginate-based hydrogels for the sustained release of quercetin	Versatile delivery system for single and combination therapies	[49]
Multifunction hydrogel	Alginate-based hydrogels for the sustained release of curcumin	Versatile delivery system for single and combination therapies	[50]

**Table 2:** Applications of carrageenan-based hydrogels.

Application	Carrageenan-Based Formulation	Benefits	References
Multifunction hydrogel	Alginate/carrageenan microgels delivering natural immunoglobulins	Improved pharmacokinetics and pharmacological action	[66]
Multifunction hydrogel	Carrageenan/pectin gels delivering natural enzymes (β-galactosidase)	Decreased enzyme sensitivity and increased compound stability during digestion	[67]
Multifunction hydrogel	pH-responsive hydrogel beads based on alginate, κ-carrageenan, and Poloxamer delivering curcumin	Enhanced encapsulation and controlled release efficiency	[68]
Multifunction hydrogel	Hydrogel composed of κ-carrageenan-containing nanocomplexes able to encapsulate blueberry anthocyanins	Safeguarded against anthocyanin degradation at low pH	[70]
Colon-targeted device	Whey protein/κ-carrageenan hydrogels delivering curcumin	Preserved curcumin from degradation during digestion	[71]
Hydrogel beads	Hydrogel beads made of cross-linked carrageenan and alginate delivering curcumin	Enhanced drug solubility, metabolic stability, non-toxicity, and capacity to target cancer cells more specifically	[72]

**Table 3:** Applications of chitosan-based hydrogels.

Application	Chitosan-Based Formulation	Benefits	References
Dressing for wound healing	Chitosan-based hydrogel containing phloroglucinol	Improved antioxidant and antimicrobial activities	[81]
Dressing for wound healing	Chitosan/silk hydrogel loaded with purified polysaccharide from Curcuma zedoaria and platelet-rich plasma–exosomes	Improved antioxidant and antimicrobial activities	[82]
Treatment of excisional wounds	Chitosan-based gel containing vitexin	Improved antioxidant, anti-inflammatory, antiviral, and antibacterial properties	[83]
Dressing for wound healing	Chitosan hydrogel incorporating Fagonia indica	Improved skin wound re-epithelialization and wound healing abilities	[84]
Functionalized platform for wound reparation	Cross-linked chitosan/gelatin hydrogel containing polyphenolic extract from Hamamelis virginiana	Inhibition of metalloproteases and reactive oxygen species Antibacterial activity against P. aeruginosa and S. aureus	[85]
Dressing for chronic wound healing	Soft hydrogel based on modified chitosan containing P. granatum peel extract	Improved antioxidant and antimicrobial activities	[86]
Dressing for acute wound healing	Biocompatible hydrogel film embedding Periplaneta americana extract	Improved antioxidant and antimicrobial activities	[87]
Dressing for wound healing	Chitosan-based hydrogels containing aloe vera juice	Regenerative effect on skin wounds; anti-inflammatory and antibacterial effect	[88]
Dressing for wound healing	Chitosan-based hydrogels containing aloe vera gel	Regenerative effect on skin wounds; anti-inflammatory and antibacterial effect	[89]
Dressing for wound healing	Bee chitosan-based hydrogels modified with yellow tea extract	Regenerative effect on skin wounds; anti-inflammatory and antibacterial effect	[90]
Dressing for wound healing	Bletilla striata polysaccharide/carboxymethyl chitosan/carbomer 940 hydrogel	Regenerative effect on skin wounds; anti-inflammatory and antibacterial effect	[91]
Dressing for wound healing	Chitosan-based hydrogel containing Salvia officinalis	Regenerative effect on skin wounds; anti-inflammatory and antibacterial effect	[92]
Dressing for wound healing	Ethyl acetate Salix alba leaf extract-loaded chitosan-based hydrogel film	Regenerative effect on skin wounds; anti-inflammatory and antibacterial effect	[93]
Dressing for wound healing	Lawsonia inermis ethanolic extract loaded in chitosan/PVA hydrogel	Improved antioxidant and antimicrobial activities	[94]
Dressing for wound healing	Essential oil-rich copaiba loaded in chitosan-based hydrogel	Acceleration in wound healing and optimal antibacterial and anti-inflammatory effect	[95]
Dressing for wound healing	Essential oil from Eucalyptus, Ginger, and Cumin loaded in chitosan-based hydrogel	Acceleration in wound healing and optimal antibacterial and anti-inflammatory effect	[96,97]
Dressing for wound healing	Bee honey incorporated into chitosan-based hydrogel	Maintenance of well-structured epidermis and acceleration of wound healing	[98]
Gel for bone strengthening	Chitosan-coated semi-interpenetrating polymer hydrogels, containing sodium alginate and poly(2-ethyl-2-oxazoline) loaded with Thymol	Stimulation of osteoblastic differentiation without cytotoxicity on mesenchymal stem cells	[99]
Gel for bone disease treatment	Calcium carbonate microcapsules containing nanohydroxyapatite/chitosan/collagen hydrogel particles containing quercetin	Prolonged-release profiles of flavonoid; promotion of bone regeneration of bones	[100]
Hydrogel to reduce joint edema and advancement of arthritis and bone erosion	Phenethylisothiocyanate loaded in an injectable chitosan/pluronic hydrogel	Improvement of bioactive’s water solubility and half-life	[101]
In situ gelling device for the treatment of spinal cord lesions	In situ gelling chitosan hydrogels delivering cannabidiol	Prolonged release	[102]
Gel for treatment of periodontitis	Chitosan hydrogel loading caffeic acid phenyl ester	Effective reduction in inflammation and simultaneous repair of bone tissue	[104]
Gel for treatment of dental decay	Chitosan hydrogel encapsulating curcumin	Inhibitory effects against Streptococcus mutants	[105]
Antibacterial device	Turmeric cyclodextrin-grafted chitosan hydrogel	Antimicrobial activity against Staphylococcus aureus and E. coli	[106]
Antimicrobial device	Chitosan nanohydrogels loaded with tanshinone	Antibacterial and anti-biofilm activity against Streptococcus	[107]
Gel used as antineuroinflammatory agent	Rhein–chitosan hydrogel	Improved mechanical strength, sustained release, and low toxicity characteristics	[108]
Gel used for neuritis treatment	Chitosan gel containing Aster glehni leaf extract	Controlled release	[109]
Multifunction nanohydrogel	Chitosan nanohydrogels containing Yerba mate	Prolonged release of antioxidant compounds	[110]
Gel for acne treatment	Chitosan hydrogel containing tea tree oil for acne treatment	Improved antimicrobial activity	[111]
pH-sensitive delivery platform for anticancer purposes	Chitosan gel containing carvacrol	Controlled release	[112]
pH-sensitive delivery platform for anticancer purposes	Chitosan gel containing quercetin	Controlled release	[113]
pH-sensitive delivery platform for anticancer purposes	Chitosan-based hydrogel containing resveratrol	Rapid absorption into gastric fluid	[114]
pH-sensitive delivery platform for anticancer purposes	Chitosan-coated alginate microbeads containing leaf extract of Moringa oleifera	Improved drug stability	[115]

**Table 4:** Applications of agarose-based hydrogels.

Application	Agarose-Based Formulation	Benefits	References
Multifunction hydrogel	Agarose nanostructured aerogels delivering natural substances	Improved drug delivery characteristics	[127]
Anticancer hydrogel	Nanocomposite containing chitosan, agarose, and graphitic carbon nitride to load and release curcumin	Enhanced encapsulation and controlled release efficiency.	[130]
Multifunction hydrogel	Flexible pH-responsive agarose/succinoglycan hydrogels	Controlled drug release	[131]
Multifunction nanocomposite hydrogel	pH-responsive hydrogel nanocomposite made from agarose, polyvinylpyrrolidone, and hydroxyapatite loading quercetin	Improved loading capacity, sustained release, and apoptosis-inducing actions	[132]

**Table 5:** Applications of hyaluronic acid-based hydrogels.

Application	Hyaluronan-Based Formulation	Benefits	References
pH-sensitive hydrogel for transdermal drug delivery	pH-sensitive hydrogel containing isoliquiritigenin using hydroxyethyl cellulose and hyaluronic acid	pH-dependent drug release, with higher drug release at pH levels above 7	[141]
Composite hydrogel for wound healing	Composite hydrogel incorporating tannic acid and dopamine-coated carbon particles rich in phenols	Rapid gelation time, exceptional adhesive strength, low hemolytic activity, minimal cytotoxicity, and ability to promote fibroblast proliferation and migration	[142]
Hydrogel for the treatment of lower limb skin wound in patients with diabetes mellitus	Hyaluronic acid-based nanohydrogel embedded with quercetin and oleic acid	Reduction in wound healing time without developing adverse drug reactions	[143]
Hydrogel for localized and sustained release in periodontal pockets	Opuntia ficus-indica extract encapsulated in chitosan nanoparticles embedded in pluronic–hyaluronic thermo-responsive hydrogel	System able to eradicate biofilms of S. mutans, P. aeruginosa, and P. gingivalis and disrupt extracellular polymeric substance formation.	[144]
Hydrogel for treatment of atopic dermatitis	Hyaluronic acid hydrogel containing resveratrol-loaded chitosan (CS) nanoparticles	Delayed hydrolytic degradation and slowed resveratrol release. Decreased oxidative damage in TNF-α/INF-γ-treated human keratinocytes (HaCaTs) and reduced secretion and gene expression of proinflammatory cytokines	[19]
Localized drug delivery platform	Hydroxytyrosol-loaded chitosan nanoparticles in an in situ hydrogel composed of pluronic F-127 and hyaluronic acid	Reduced oxidative and inflammatory effects in chondrocyte cellular model and influence on chondrocyte gene expression under pathological state	[20]
